# Cognitive and Autonomic Dysfunction in Multiple System Atrophy Type P and C: A Comparative Study

**DOI:** 10.3389/fneur.2022.912820

**Published:** 2022-06-16

**Authors:** Giulia Lazzeri, Giulia Franco, Teresa Difonzo, Angelica Carandina, Chiara Gramegna, Maurizio Vergari, Federica Arienti, Anisa Naci, Costanza Scatà, Edoardo Monfrini, Gabriel Dias Rodrigues, Nicola Montano, Giacomo P. Comi, Maria Cristina Saetti, Eleonora Tobaldini, Alessio Di Fonzo

**Affiliations:** ^1^Neurology Unit, Fondazione IRCCS Ca' Granda, Ospedale Maggiore Policlinico, Milan, Italy; ^2^Centro Dino Ferrari, Neuroscience Section, Department of Pathophysiology and Transplantation, University of Milan, Milan, Italy; ^3^Department of Internal Medicine, Fondazione IRCCS Ca' Granda, Ospedale Maggiore Policlinico, Milan, Italy; ^4^Department of Clinical Sciences and Community Health, University of Milan, Milan, Italy; ^5^PhD Program in Neuroscience, School of Medicine and Surgery, University of Milano-Bicocca, Monza, Italy; ^6^Neurophysiology Unit, Fondazione IRCCS Ca' Granda, Ospedale Maggiore Policlinico, Milan, Italy; ^7^Department of General Psychology, University of Padua, Padua, Italy

**Keywords:** multiple system atrophy, cognitive dysfunction, neuropsychological assessment, dysautonomia, heart rate variability

## Abstract

Multiple System Atrophy (MSA) is a rare neurodegenerative disease, clinically defined by a combination of autonomic dysfunction and motor involvement, that may be predominantly extrapyramidal (MSA-P) or cerebellar (MSA-C). Although dementia is generally considered a red flag against the clinical diagnosis of MSA, in the last decade the evidence of cognitive impairment in MSA patients has been growing. Cognitive dysfunction appears to involve mainly, but not exclusively, executive functions, and may have different characteristics and progression in the two subtypes of the disease (i.e., MSA-P and MSA-C). Despite continued efforts, combining *in-vivo* imaging studies as well as pathological studies, the physiopathological bases of cognitive involvement in MSA are still unclear. In this view, the possible link between cardiovascular autonomic impairment and decreased cognitive performance, extensively investigated in PD, needs to be clarified as well. In the present study, we evaluated a cohort of 20 MSA patients (9 MSA-P, 11 MSA-C) by means of a neuropsychological battery, hemodynamic assessment (heart rate and arterial blood pressure) during rest and active standing and bedside autonomic function tests assessed by heart rate variability (HRV) parameters and sympathetic skin response (SSR) in the same experimental session. Overall, global cognitive functioning, as indicated by the MoCA score, was preserved in most patients. However, short- and long-term memory and attentional and frontal-executive functions were moderately impaired. When comparing MSA-P and MSA-C, the latter obtained lower scores in tests of executive functions and verbal memory. Conversely, no statistically significant difference in cardiovascular autonomic parameters was identified between MSA-P and MSA-C patients. In conclusion, moderate cognitive deficits, involving executive functions and memory, are present in MSA, particularly in MSA-C patients. In addition, our findings do not support the role of dysautonomia as a major driver of cognitive differences between MSA-P and MSA-C.

## Introduction

Multiple system atrophy (MSA) is a rare pleiotropic neurodegenerative disease, belonging to the broad family of α-synucleinopathies. Its clinical hallmark is the combination of progressive autonomic dysfunction with extrapyramidal and/or cerebellar signs, depicting two distinct motor phenotypes, MSA-P and MSA-C (1). Like other α-synucleinopathies, namely Parkinson's Disease (PD) and Dementia with Lewy Bodies (DLB), the clinical spectrum of MSA encompasses a wide range of non-motor symptoms (NMS), including behavioral and cognitive involvement. Dementia, as defined by the DSM-IV criteria, is a non-supporting feature in the current clinical diagnostic criteria for MSA (2). Nevertheless, the evidence of cognitive impairment among MSA patients has been growing over the years. In fact, dementia may be present in up to 15% of neuropathologically proven MSA patients, generally appearing in the later phases of the disease (3, 4). Moreover, about one-third of patients present a mild cognitive impairment even in the initial stages (5), particularly affecting executive functions, but possibly involving other domains, like memory, visuospatial, and constructional functions (6–8). Given such a high frequency of cognitive involvement, neuropsychological assessment should be a part of the diagnostic work-up of MSA patients. Comparative studies on cognitive functions in MSA-P and MSA-C have observed controversial results and the neuropsychological profile of these subsets of patients remains poorly characterized (9, 10).

Neuropathological findings and *in-vivo* imaging studies suggest the hypothesis that cognitive decline in MSA originates from frontostriatal dysfunction, however cortical and cerebellar degeneration may further contribute to the decline. Patients with cognitive decline have also been shown to have a greater burden of neuronal cytoplasmic inclusions (NCI) in the dentate gyrus in neuropathologic assessment, compared with patients without cognitive decline (7).

The possible role of cardiovascular dysautonomia in the development of MSA cognitive decline, suggested by previous studies in PD, remains to be clarified. Interestingly, in a systematic review, a possible association between orthostatic hypotension (OH) and cognitive impairment in α-synucleinopathies has been illustrated, even if the underlying mechanisms are unclear (11). While some authors argued that cardiovascular dysautonomia is a risk factor for cognitive impairment in MSA (5, 12), the clinicopathological study by O'Sullivan et al. (4) did not confirm this link, being autonomic dysfunction a predictor of shorter time to death, but not of cognitive dysfunction.

Even though dysautonomic manifestations are a cardinal clinical feature of the disease and are regularly investigated during the neurological examination, the neurophysiological and cardiovascular techniques assessing the autonomic nervous system (ANS), like tilt table testing, are not easily accessible in daily clinical routine. On the other hand, the active standing test is a simple test to be performed at the bedside or in an outpatient setting in order to assess the cardiovascular autonomic control (13, 14) and several bedside autonomic assessments have been developed in the last years, with possible simpler use in daily clinical practice. Among them, heart rate variability (HRV), the variation in time intervals between heartbeats, is a non-invasive and sensitive indicator of autonomic alterations. and it can represent a measure of the global ability of the ANS to adapt to endogenous and external stimuli, with a particular value in neurological disorders involving the ANS, including PD and MSA (15). Similarly, Sympathetic Skin Response (SSR) is an easily obtainable index of sympathetic sudomotor function and is defined as the momentary change of the electrical potential of the skin (16), and has been used as a possible marker of autonomic dysfunction in both PD (17, 18) and MSA (19).

The present study aims to assess the cognitive performances of patients suffering from either MSA-P or MSA-C and their cardiovascular and cutaneous autonomic function, in the same experimental session, and subsequently explore a potential link between the cognitive profile and dysautonomia.

## Materials and Methods

### Study Design and Population

Patients fulfilling the diagnosis of either probable MSA-P or MSA-C, according to current criteria (2), assessed at the Movement Disorders outpatient clinic of Fondazione IRCCS Ca' Granda Ospedale Maggiore Policlinico between December 2021 and January 2022 were included. For all patients, available brain Magnetic Resonance Imaging (MRI) was consistent with the clinical diagnosis of MSA.

The absence of stable sinus rhythm on ECG, history of coronary artery disease, the presence of cardiac stimulators, onco-hematological conditions, ongoing acute clinical conditions, and consent refusal were considered exclusion criteria. Every patient underwent a single assessment: each experimental session consisted of a neurologic evaluation, a 20 min ECG, blood pressure and respiratory monitoring. Subsequently, a complete neuropsychological assessment was performed. All the experimental sessions took place between 8 a.m. and 1 p.m. The protocol was approved by the local Ethics Committee (Comitato Etico Milano Area 2, approval document number: 1253_2021) and it was developed in accordance with the Declaration of Helsinki. All the subjects signed informed written consent prior to study participation.

### Clinical Evaluation

All patients underwent a complete neurologic evaluation, performed by a Neurologist trained in Movement Disorders. They were staged with Hohen and Yahr (H&Y) scale (20). The Unified Multiple System Atrophy Scale (UMSARS) was administered to evaluate disease severity (21). For each enrolled patient, the following information was collected: age at evaluation and at onset of motor symptoms, sex, disease duration, years of education, presence of non-motor symptoms (RBD, constipation, urinary disturbs) and of cardiovascular comorbidities, use of L-Dopa, L-Dopa daily dose, use of dopamine agonists, L-Dopa equivalent daily dosage (LEDD) (22, 23), use of antihypotensive drugs.

### Physiological Recordings and Cardiovascular Autonomic Control Assessment

Cardiovascular recordings were performed with spontaneous breathing in the supine position for 10 min and during active standing for 10 min. All participants were asked to avoid consuming food, caffeine, anti-hypertensive and anti-hypotensive drugs in the 2 h before the recording session and carrying out physical exercise the day before. ECG (lead II) and respiration through a thoracic piezoelectric belt were recorded with a sampling frequency of 250 Hz, using an *ad hoc* telemetric system device (Equivital, ADInstruments, Sydney, Australia).

Intermittent arterial blood pressure was measured with a validated automated cuff sphygmomanometer over the brachial artery in supine position after 5 and 10 min and 3 and 5 min after assuming the orthostatic position. Intermittent heart rate (HR) values were also recorded at the same time points. The mean values of blood pressure and HR in supine position were considered for the baseline assessment. The drop in systolic blood pressure (SBP) and diastolic blood pressure (DBP) was calculated at the third minute of active standing through the function ΔSPB/ΔDBP = (BP_SUP_ - BP_3′ORT_). Changes in HR between clinostatism and the third minute of active standing were also evaluated.

All measurements were performed in a quiet and temperature-controlled room (between 22 and 24°C), and all patients had a normal body temperature during the recordings (between 35.5 and 36.5°C).

Segments of 250 ± 50 beats were selected from each ECG signal, one at rest and one in the orthostatic position, for the analysis of HRV. Non-linear symbolic analysis was applied through a specific software (Heart Scope II, AMPS, ITA) in order to assess the cardiovascular autonomic control. In pathological conditions and in presence of dysautonomia, non-linear symbolic analysis seems to be more flexible in detecting the activity of the two autonomic branches than classical HRV analysis methods such as spectral analysis. As a matter of fact, symbolic analysis was found suitable to assess non-reciprocal changes in sympathetic and parasympathetic modulation on heart period time series, especially in patients characterized by low global variability (24, 25). Furthermore, as it is focused on short patterns in the R-R interval series, this type of analysis has been proposed to be more accurate for the study of short non-linear HRV instabilities.

Thus, the R-R time series was converted into a sequence of symbols that was divided into 3-beat patterns. Patterns were classified into 4 families: (a) 0V, patterns with no variation, all 3 symbols are equal (e.g., 4-4-4); (b) 1V, patterns with 1 variation, 2 consecutive symbols are equal forming a 2-beat plateau, while the remaining one is different (e.g., 2-2-5); (c) 2LV, patterns with 2 like variations, all symbols are different from the previous one and they are in ascending or descending order (e.g., 1-3-4); (d) 2UV, patterns with 2 unlike variations, all symbols are different from the previous one but not in a consequent order (e.g., 2-5-1). The percentage of the patterns 0V is a marker of cardiac sympathetic modulation and 2UV or 2LV are markers of cardiac vagal modulation (26).

Neurogenic supine hypertension (SH) was defined, according to current guidelines, as an SBP ≥ 140 mmHg and/or DBP ≥ 90 mmHg in supine position (27). OH was defined, according to current guidelines, as an SBP fall of ≥ 20 mmHg and/or a DBP fall of ≥ 10 mmHg within 3 min of standing (28).

### Sympathetic Skin Response

SSR of bilateral upper and lower limbs was measured using a multichannel computerized electromyograph by a trained neurophysiologist. The stimulator was placed over a median nerve at the wrist with a stimulation intensity of 20 mA for 0.2 ms. Surface electrodes were placed on the palms and plants of the right limbs, with reference electrodes placed on the corresponding dorsal regions. The filter setting was 0.5–2,000 Hz. For each limb, three recordings were performed with stimulus intervals between 30 and 60 s. The widest amplitude and its corresponding latency were measured. The response was considered absent when no change larger than 50 μV could be observed in any of the three recordings in 2 s following a stimulus. Of note, only 16 patients of the total cohort could be registered.

### Neuropsychological Evaluation

All patients underwent a series of standardized and validated neuropsychological tests during a single session lasting <2 h. The neuropsychological test battery was composed of fifteen commonly used neuropsychological tests evaluating: global cognitive functioning (Montreal Cognitive Assessment) (29), attention (Attentional Matrices, Trail Making Test) (30, 31), verbal fluency (Phonemic and Semantic Fluency) (32), memory (Digit Span and Corsi Block forward and backward, Prose Memory) (30), deductive reasoning (Raven's Colored Progressive Matrices) (33), praxis (Copying of Figures) (30) and frontal lobe/executive functions (Verbal and Alternate Fluency, Frontal Assessment Battery, Paced Auditory Serial Addition Task, Stroop Test, Clock Drawing Test) (32, 34–37).

### Statistical Analysis

Data were analyzed using SPSS Statistics 27 (IBM, Armonk, New York, USA). The Shapiro-Wilk test was used to evaluate the normal distribution of the data. For descriptive analysis, results were expressed as absolute frequency, relative frequency, means and standard error. The Student *t*-test was performed to compare normally distributed cardiovascular parameters. The Wilcoxon-Mann-Whitney non-parametric test was used to compare non-normally distributed cardiovascular parameters and performances at neuropsychological tests between the two groups of patients (MSA-P vs. MSA-C). Partial correlation analyses, controlling for age, years of education and disease duration, between neuropsychological tests and clinical variables (i.e., UMSARS scales, ΔSBP, ΔDBP, and ΔHR) were also conducted for both groups. ^*^p < 0.05 was considered statistically significant.

## Results

The demographic and clinic characteristics of MSA patients are detailed in Table 1. A total of 20 MSA patients were enrolled, of those 9 had a parkinsonian phenotype and 11 a cerebellar one. Thirteen patients (65%) were male, and the mean age at evaluation was 60.3 years (±7.6).

**Table 1:** Demographic and clinical features of MSA-P and MSA-C patients.

	**Overall**	**MSA-P**	**MSA-C**	
**Characteristics**	**Mean ± Standard Deviation, Median [IQR], *n*° (%)**	**Mean ± Standard Deviation, Median [IQR], *n*° (%)**	**Mean ± Standard Deviation, Median [IQR], *n*° (%)**	***p***
*n*	20	9 (45%)	11 (55%)	
Age	60.3 ± 7.6	60.4 ± 9.1	60.2 ± 6.7	0.942
Sex (male)	13% (65%)	5 (66%)	8 (73%)	0.642
Age at onset (years)	56.5 ± 7.6	55.9 ± 7.8	57.0 ± 7.7	0.754
Disease duration (years)	3.9 ± 2.0	4.44 ± 2.2	3.45 ± 1.9	0.295
H&Y	3.20 ± 0.52	3.22 ± 0.4	3.18 ± 0.6	0.869
UMSARS Part 1	24.4 ± 6.8	26.1 ± 8.0	23.0 ± 5.7	0.325
UMSARS Part 2	25.3 ± 8.1	25.9 ± 8.9	24.7 ± 7.9	0.760
UMSARS Part 4	2.70 ± 0.9	2.66 ± 1.1	2.72 ± 0.9	0.895
Education (years)	12.68 ± 4.3	14.50 ± 3.8	11.36 ± 3.4	0.121
**NMS**				
Constipation	19 (95%)	8 (89%)	11 (100%)	0.450
Urinary incontinence	18 (90%)	9 (100%)	9 (82%)	0.479
Urinary retention	16 (80%)	7 (78%)	9 (82%)	1.000
RBD	19 (95%)	9 (100%)	10 (91%)	1.000
**Therapy**				
Total LEDD	300 [119–545]	300 [263–735]	175 [0–400]	0.022^*^
Levodopa therapy	15 (75%)	9 (100%)	6 (55%)	0.038^*^
Anti-hypotensive therapy	5 (25%)	2 (22%)	3 (27%)	1.000

*MSA-P, Multiple System Atrophy, parkinsonian type; MSA-C, Multiple System Atrophy, cerebellar type; H&Y, Hohen and Yahr scale; UMSARS, Unified Multiple System Atrophy Rating Scale; NMS, non-motor symptoms; RBD, REM sleep behavior disorder; LEDD, levodopa equivalent daily dose*.

* *Significant p-values < 0.05*.

The average age of disease onset was 56.5 years (±7.6), with a mean disease duration at evaluation of 3.9 years (±2.0), without any significant difference between MSA-P and MSA-C patients.

The two cohorts showed similar disease severity, assessed by H&Y and UMSARS scale, and comparable frequency of non-motor symptoms (NMS).

As expected, L-dopa therapy was used more frequently in MSA-P patients (*p* = 0.038).

The mean number of years of education was 12.68 (±4.3), uniform in the two groups of patients.

The results of bedside autonomic and hemodynamic tests performed in the two cohorts are outlined in Table 2.

**Table 2:** Cardiovascular and cutaneous dysautonomia evaluation in MSA-P and MSA-C patients.

	**MSA-P (*N =* 9)**	**MSA-C (*N =* 11)**	
**Characteristics**	**Mean ± Standard Deviation, Median [IQR], *n*° (%)**	**Mean ± Standard Deviation, Median [IQR], *n*° (%)**	***p***
SBP_baseline	142.8 ± 20.9	142.9 ± 26.9	0.991
DBP_baseline	82.0 [79.5–96.5]	81.0 [76.0–100.0]	0.970
HR_baseline	69.0 [64.5–82.5]	71.0 [65.0–87.0]	0.820
ΔSBP	35.4 ± 21.5	27.0 ± 17.8	0.961
ΔDBP	15.9 ± 11.7	14.9 ± 11.2	0.980
ΔHR	7.6 ± 7.2	8.8 ± 10.1	0.756
SH	4 (44%)	4 (36%)	1.000
OH	8 (89%)	7 (64%)	0.221
SH+OH	4 (44 %)	3 (27%)	0.642
**HRV parameters at rest**			
0V%	23.5 [16.5–34.6]	28.5 [25.4–34.1]	0.648
2LV%	5.8 ± 4.0	4.1 ± 2.9	0.290
2UV%	23.8 ± 11.2	26.3 ± 10.2	0.612
**SSR**			
**[** ***16 patients*** **]**			
Hand (no response)	42.9% (3)	33.3% (3)	1.000
Foot (no response)	42.9% (3)	44.4% (4)	1.000

*MSA-P, Multiple System Atrophy, parkinsonian type; MSA-C, Multiple System Atrophy, cerebellar type; SBP, systolic blood pressure; DBP, diastolic blood pressure; SH, supine hypertension; ΔSBP, reduction in systolic blood pressure in response to active standing test; ΔDBP, reduction in diastolic blood pressure in response to active standing test; ΔHR, increase in heart rate in response to active standing test; OH, orthostatic hypotension; HRV, heart rate variability; 0V%, patterns 0 variations; 2LV% patterns with 2 like variations; 2UV%, pattern with 2 unlike variations*.

No statistically significant differences in arterial blood pressure at rest and in arterial blood pressure changes in response to the active standing test were identified between MSA-P and MSA-C patients. OH was present in a majority of patients in both subsets (respectively, 89 and 64%, *p* = 0.221), while SH was present in less than half of the cases, even though mean values of both SBP and DBP were just above the cut-off values for the definition of SH.

Symbolic HRV parameters at rest did not differ significantly in the two subgroups, considering both sympathetic (0V%) and parasympathetic (2LV% and 2UV%) modulation.

SSR was abnormal in similar proportions in the two subgroups, in both sites of registration (hand and foot).

Table 3 presents the scores obtained at neuropsychological tests by MSA-P and -C patients. Overall, MoCA scores were within normal limits, with only one person reaching a borderline score, according to updated norms (38).

**Table 3:** Cognitive performances of MSA-P and MSA-C patients at neuropsychological tests.

**Test**	**Mdn ± IQR (MSA-P)**	**Mdn ± IQR (MSA-C)**	***p***
MoCA	27 ± 4.5	24 ± 7	0.135
Attentional Matrices	52 ± 9	45 ± 20	0.045^*^
TMT-A	43.5 ± 27.3	57.5 ± 44	0.230
TMT-B	101 ± 51	134.5 ± 131	0.045^*^
TMT-B-A	50 ± 47	68 ± 72.5	0.035^*^
RCPM	31 ± 5.3	33 ± 9	0.836
Phonemic Fluency	34.5 ± 28	31.5 ± 26	0.328
Semantic Fluency	45 ± 19.5	41.5 ± 11.5	0.563
Alternate Fluency	29 ± 15.5	25 ± 23	0.688
Composite Shifting Score	0.7 ± 0.2	0.8 ± 0.2	0.350
DS-Forward	5 ± 1.8	6 ± 3	0.410
DS-Backward	4 ± 1	4 ± 2	0.386
CB-Forward	5.5 ± 1	5 ± 2	0.576
CB-Backward	4 ± 1	4 ± 1	0.892
Prose Memory	16 ± 3.8	13.3 ± 6.9	0.196
Copying of Figures	14 ± 1	13 ± 2.25	0.167
CDT	13 ± 1	12 ± 1	0.029^*^
FAB	17.5 ± 1.8	15 ± 3	0.034^*^
Stroop Test	26.2 ± 17.5	20 ± 12.8	0.285
PASAT	33 ± 24	33 ± 20.5	0.958

*MoCA, Montreal Cognitive Assessment; TMT, Trail Making Test; RCPM, Raven's Colored Progressive Matrices; DS, Digit Span; CB, Corsi Block; CDT, Clock Drawing Test; FAB, Frontal Assessment Battery; PASAT, Paced Auditory Serial Addition Test*.

* *Significant p-values < 0.05*.

The most affected cognitive domains, with abnormal scores in 10 to 25% of patients, were short- and long-term memory (Digit Span, Corsi block, Prose Memory) and especially attentional and frontal-executive functions (Trail Making Test, Attentional Matrices, Verbal Fluency, Clock Drawing Test, FAB and PASAT).

When comparing MSA-C and MSA-P patients, we found significant differences in the performances at the following tests: Attentional Matrices (*U* = 16.5, *p* = 0.045), Trail Making Test part B (*U* = 14.5, *p* = 0.045) and B-A (*U* = 13.5, *p* = 0.035), Clock Drawing Test (*U* = 16, *p* = 0.029) and Frontal Assessment Battery (*U* = 19, *p* = 0.034). Notably, MSA-C patients obtained lower scores in all the above-mentioned tests, as shown in Table 3.

To conduct a partial correlation analysis between the neuropsychological scores and measured clinical variables, we grouped the neuropsychological tests administered into the following three categories: attentive-executive (Attentional Matrices, Trail Making Test, Alternate Fluency, Digit Span backward, Corsi Block backward, Frontal Assessment Battery, Paced Auditory Serial Addition Task, Stroop Test, and Clock Drawing Test), memory (Digit Span forward, Corsi Block forward and Prose Memory) and visuospatial (Raven's Colored Progressive Matrices, Corsi Block forward and backward, Copying of Figures, Clock Drawing Test). Almost no significant correlations were found between the three categories of neuropsychological tests and clinical variables, including autonomic functioning, as expressed by ΔSBP, ΔDBP, and ΔHR upon standing (all > *p* = 0.05). as shown in Table 4. There was only one negative association between the scores obtained at neuropsychological tests involving visuospatial abilities and ΔDBP in MSA-P patients.

**Table 4:** Partial correlations between clinical variables and scores obtained at neuropsychological tests (divided by cognitive function).

**Clinical variables**	**Cognitive functions**					
	**MSA-P**			**MSA-C**		
	**A-E**	**M**	**VS**	**A-E**	**M**	**VS**
UMSARS Part 1	0.204	0.563	−0.334	−0.187	−0.628	−0.056
UMSARS Part 2	0.452	0.616	−0.634	0.355	0.232	−0.066
UMSARS Part 4	0.405	0.319	−0.503	0.420	−0.041	−0.232
ΔSBP	0.474	0.671	−0.621	0.796	0.651	0.555
ΔDBP	0.872	0.875	−0.965^*^	0.469	0.408	0.289
ΔHR	−0.047	0.510	−0.136	0.387	−0.008	0.068

*A-E, Attentive-executive; M, Memory; VS, Visuospatial*.

* *Significant p-values < 0.05*.

## Discussion

The aim of this study was the evaluation, in the same experimental session of cognitive performances and autonomic dysfunction in MSA-P and MSA-C patients and to compare the results of these two groups. This is, to our knowledge, the first study assessing cognitive and autonomic functions in the same experimental session. Overall, MSA-C patients reached lower scores in tests of executive functions and verbal memory, no statistically significant difference in cardiovascular autonomic parameters was identified between MSA-P and MSA-C patients.

On the one hand, Brown et al. showed that, when confirming neuropathologically the clinical diagnosis of MSA, patients who had cognitive impairment were significantly more often misdiagnosed than patients without cognitive impairment (35.6 vs. 5.6%) (9), being the final pathologic diagnosis of misdiagnosed patients progressive supranuclear palsy, DLB and amyotrophic lateral sclerosis. On the other hand, 59% of pathologically proven MSA patients that received a different clinical diagnosis during life had cognitive impairment (7). This highlights the importance of the characterization of specific features of cognitive dysfunctions in MSA patients and their role in the differential diagnosis with other neurodegenerative disorders. Moreover, only a few studies have performed a longitudinal follow up on neuropsychological assessment in MSA patients (12, 39–41), showing significant worsening over time only in subgroups of patients, suggesting a possible individual predisposition, thus making it essential to identify possible clinical predictors for the development of cognitive impairment.

The present cohort showed a good performance of general cognitive functioning, as assessed with MoCA. However, we found that 40% of the patients met the mild cognitive impairment (MCI) diagnostic criteria (42). Indeed, even in the absence of dementia, we found impaired or borderline scores in different cognitive domains, especially in attentional, memory and frontal-executive ones. This result matches those observed in earlier studies that have shown mild cognitive deficits in MSA patients (7). Other studies have used Mini Mental State Examination (MMSE) as a screening tool for cognitive impairment in MSA patients, finding it abnormal generally in less than a quarter of patients (5, 43). FAB was abnormal in 10% of patients, slightly lower than what was previously found in other cohorts (ranging from 32 to 42%) (5, 43–45).

The comparison of the two groups at neuropsychological tests showed that MSA-C patients had worse performances at executive tests than MSA-P. This prominent executive dysfunction is manifested by impairment in mental flexibility, attentional set-shifting, automatic response generation, abstract thinking and planning. The first studies assessing cognition in MSA patients were mostly focusing on the MSA-P subgroup, compared to PD patients, describing frequently mild to moderate deficits in executive functions, attention and phonemic fluency, and less frequent involvement of semantic fluency and visuospatial functions (46–52). Others described MSA-C patients' features, again underlining the presence of frontal-executive dysfunction (53, 54). Since 2008, with the study by Kawai et al. numerous attempts have been made to dissect the peculiar cognitive profiles of the parkinsonian and cerebellar phenotypes, with discordant results. Some could find no significant difference between the two cohorts (7, 12, 43, 55, 56), while others outlined a more severe and widespread cognitive dysfunction in MSA-P patients on neuropsychological batteries (9, 57, 58), but also when screening very large numbers of patients with MoCA (59). Nevertheless, results from other groups seem in line with our findings. For instance, Eschlböck et al. (45) found that MSA-C patients performed significantly worse than MSA-P in executive functions, screened with FAB and in phonological verbal fluency, confirming previous reports of a predominance of attentional and executive deficits in MSA-C (10, 60). The performances of the two subgroups may also be affected by different frequencies of depression and anxiety (61). Interestingly, Santangelo et al. in a recent longitudinal report, identify possible differences in the evolution of cognitive dysfunction in the two subtypes, with a significant worsening in prose memory, spatial planning, repetition abilities and functional autonomy in MSA-P, and on the other hand a more severe deterioration in a less widespread fashion, mainly in cognitive tests assessing spatial planning and psychomotor speed in MSA-C (55). This finding was not identified in a previous report on a smaller cohort (41). Thus, the possible divergence of the cognitive decline depending on the motor phenotype has to be confirmed in future prospective studies on a larger number of patients. If confirmed, one may speculate that this discrepancy may be due to differences in the topographic progression of the neurodegeneration in the two motor phenotypes.

To date, the pathogenic bases of the cognitive impairment found in MSA patients still need to be clarified. The general concept of “subcortical dementia” may somewhat explain cognitive features of MSA, with a disruption of striato–pallido–thalamocortical circuits due to the prominent degeneration of substantia nigra and putamen (62). Neuropathologic studies have also identified prominent cortical degeneration, especially in frontal lobes (63, 64). 18F-fluorodeoxyglucose PET imaging studies have suggested a possible correlation between the severity of cognitive impairment in MSA-P patients and hypoperfusion in the dorsolateral prefrontal cortex (9), and hypometabolism in the frontal gyrus and cerebellum may correlate with the severity of cognitive impairments in attention, executive function, and language domains in both subtypes (58). Moreover, frontal atrophy detected by MRI in MSA patients with cognitive impairment compared with those without cognitive impairment has been described (65), and memory scores may correlate with prefrontal lobe atrophy (10). Gray matter volume measurement analysis assessed voxel-based morphometry suggests an even more precise correlation between cognitive performances and involvement of specific cortical and subcortical areas: in particular, Dash et al. recently reported an association between executive dysfunction, attention, and verbal working memory and reduced gray matter volumes in the frontal lobe, insula, and thalamus, of new learning with right superior and middle frontal gyrus volumes, immediate and delayed recall with temporal lobe, cingulate gyrus, caudate and cerebellum volumes (56). If confirmed in larger cohorts, these findings may help have a more precise description of the anatomic correlates of cognitive dysfunction in the two phenotypes. A central role of the cerebellum in cognition has been dissected over the years (66), and a significant correlation between cerebellar atrophy and cognitive dysfunction has been demonstrated in both MSA-C (67) and MSA-P (68). As a matter of fact, cerebellar output has been proved to influence the activity of prefrontal cortex and anterior cingulate cortex through thalamic connections, contributing to the regulation of flexible control, post-error processing and working memory. The precocious disruption of the cerebello-thalamo-cortical network in MSA-C patients may explain the discrepancy in executive function scores compared to MSA-P cases (62). Interestingly, a recent study dissecting the motor and non-motor clinical characteristics abstracting from the classical parkinsonian/cerebellar phenotype, suggests the existence of a third possible phenotype, with a prominent cognitive and cerebellar involvement, with no extrapyramidal nor axial manifestations (69).

HRV has been frequently used to assess autonomic function in MSA, but using mainly spectral power analysis (70–72). In the present study, we focused on the more innovative non-linear symbolic analysis, that, to the best of our knowledge, was not previously used to dissect autonomic dysfunction in MSA-P and MSA-C. Overall, we could not find significant differences in the autonomic modulation as assessed by HRV and SSR and in hemodynamic response to active standing between MSA-P and MSA-C. Different from what was described in our cohort, in which parkinsonian and cerebellar patients showed similar frequency of autonomic dysfunction, previous reports suggest possible different autonomic profiles in the two phenotypes. Wenning et al. suggested a more frequent prevalence of OH, screened by active standing, in MSA-C patients (14). This finding, although not subsequently confirmed by other groups (73–75) gave the first hint that the prominent brainstem and cerebellar involvement have an impact on the BP response to standing in MSA-C patients. Moreover, the severity of BP drop may be a predictor of death in male MSA-C patients (76) and not in the parkinsonian variant.

Interestingly, in a recent prospective cohort study, Foubert-Samier et al. did not identify significant differences in autonomic function between MSA-C and MSA-P patients in the first timepoint evaluation, but a difference was detectable after 5 years from the initial evaluation, with MSA-C patients presenting a slight worsening of OH (77). Therefore, since the mean disease duration of our cohort was 3.9. years (±2.0), differences in autonomic control and hemodynamic response to active standing between MSA-C and P may be under-detected due to the short disease duration.

Scarce evidence on the possible differences in SSR is available. Some reports suggest that abnormalities of SSR may be more frequent in MSA-C patients (78); however, we were not able to replicate this finding in our small cohort.

The possible association between cognitive dysfunction and OH has been extensively assessed in PD, suggesting a possible positive correlation, particularly in terms of attention as well as visual and verbal memory (79–81). Moreover, autonomic dysfunction has been identified as a strong predictor of the development of dementia in PD patients (82). However, the causative role of autonomic dysfunction in the development of cognitive impairment is yet to be demonstrated, since they could be a manifestation of parallel neurodegenerative processes with diffuse neuropathological involvement, or on the other hand chronic hypoperfusion and small vessel disease due to OH and SH may be at least partially responsible for cognitive decline (11, 83). On the other hand, reports about the association of autonomic dysfunction and cognitive impairment in MSA patients are less copious and with conflicting results. Cardiovascular dysautonomia has been shown to be a predictor of cognitive impairment in MSA patients as well (5). Some reports propose a possible association of coexisting OH and frontal lobe dysfunction (84), while others depict similar performances in patients with and without OH (45, 60) or find no correlation between cognition and the presence or the severity of OH (44, 85). Interestingly, the presence of OH could be associated, rather than cognitive impairment, with a greater tendency to deteriorate in longitudinal follow-up (40). The clinicopathologic study by O'Sullivan et al. found no difference in time needed to develop cognitive impairment in MSA with early vs. late autonomic symptoms (4), suggesting a different temporal evolution of cognitive and autonomic dysfunction. In the present study, we could identify only a negative association between the scores obtained at neuropsychological tests involving visuospatial abilities and ΔDBP in MSA-P patients. This result needs replication in larger cohorts to permit further interpretation. Moreover, comprehensive bedside autonomic assessment, including SSV and HRV that are not usually included in the neurological workout, could reveal unexpected correlations across broader patient cohorts, thus contributing to a better definition of the clinical heterogeneity of MSA patients.

Several limitations of the present study need to be underlined, first of all, the small sample size. All consecutive patients with probable MSA visiting the Movement Disorders outpatient clinic in a short period of time were enrolled. This may have led to a selection bias, because patients at the most advanced stages of the disease, or already living in residential care structures, come rarely to consultation in person. This is partially confirmed by the mean H&Y stage of the cohort (3.2 ± 0.5) and may have an impact on the detection of cognitive impairment since it has been reported that cognitive dysfunction occurs more commonly in the advanced stages of disease (5). Moreover, the relatively short disease duration since the onset of motor symptoms (mean 3.9 ± 2.0) may also have led to an underdetection of cognitive impairment, since it has been described to become clinically significant on average about 7 years after diagnosis (4). Another limitation is the lack of information about possible neuropsychiatric involvement, particularly depression, anxiety, and apathy, that may have had an impact on the performances of patients, especially in the executive domain.

An inevitable drawback of the study is the lack of neuropathological diagnosis, which may have lead to misdiagnosis in a certain number of patients. However, all patients carried a clinical diagnosis of probably MSA, and their mean disease duration is almost 4 years, thus reasonable to obtain a correct clinical diagnosis. Moreover, the MRIs of all patients were reviewed by experienced neuroradiologists to confirm the presence of neuroradiological findings compatible with the diagnosis.

Despite some drawbacks, this study confirms previous findings of the variable spectrum of cognitive impairments in MSA, with different profiles depending on the motor phenotype. While the global cognitive performances, as indicated by the MoCA score, remains preserved in most patients, deficits involving executive functions and verbal memory could be observed, particularly in MSA-C patients. Further prospective studies, with more copious cohorts and with longitudinal assessments, are needed to understand whether MSA-P and MSA-C may have distinctive patterns of cognitive involvement, and elucidate the possible role of concomitant dysautonomia.

## Data Availability Statement

The raw data supporting the conclusions of this article will be made available by the authors, without undue reservation.

## Ethics Statement

The studies involving human participants were reviewed and approved by Comitato Etico Milano Area 2. The patients/participants provided their written informed consent to participate in this study.

## Author Contributions

GL, GF, TD, AC, MV, FA, and AN performed patient's assessment. GL, GF, TD, AC, FA, CG, CS, EM, and GDR performed statistical analysis and correlation. GL, GF, TD, AC, NM, GC, MCS, ET, and AD design of the study and writing the manuscript. All authors contributed to the article and approved the submitted version.

## Funding

This work was supported by the Italian Ministry of Health (Ricerca Corrente 2022).

## Conflict of Interest

The authors declare that the research was conducted in the absence of any commercial or financial relationships that could be construed as a potential conflict of interest.

## Publisher's Note

All claims expressed in this article are solely those of the authors and do not necessarily represent those of their affiliated organizations, or those of the publisher, the editors and the reviewers. Any product that may be evaluated in this article, or claim that may be made by its manufacturer, is not guaranteed or endorsed by the publisher.
